# Evaluation of spatial spreading of phyto-available sulphur and micronutrients in cultivated coastal soils

**DOI:** 10.1371/journal.pone.0258166

**Published:** 2021-10-04

**Authors:** A. K. Shukla, S. K. Behera, R. Tripathi, C. Prakash, A. K. Nayak, P. Suresh Kumar, T. Chitdeshwari, Dileep Kumar, R. K. Nayak, P. Surendra Babu, R. N. Katkar, C. T. Subbarayappa, K. C. Moharana, A. K. Patra, Ch. Srinivasarao, S. K. Chaudhari, A. Subba Rao, A. K. Singh, Soumitra Das

**Affiliations:** 1 ICAR- Indian Institute of Soil Science, Bhopal, Madhya Pradesh, India; 2 ICAR-National Rice Research Institute, Cuttack, Odisha, India; 3 Kerala Agricultural University, Thrissur, Kerala, India; 4 Tamil Nadu Agricultural University, Coimbatore, Tamil Nadu, India; 5 Anand Agricultural University, Anand, Gujarat, India; 6 Odisha University of Agriculture & Technology, Bhubaneswar, Odisha, India; 7 PJTS Agricultural University, Rajendranagar, Hyderabad, Telangana, India; 8 Dr. Panjabrao Deshmukh Krishi Vidyapeeth, Akola, Maharashtra, India; 9 University of Agricultural Sciences, Bangalore, Karnataka, India; 10 ICAR-National Academy of Agricultural Research Management, Hyderabad, Telangana, India; 11 Indian Council of Agricultural Research, New Delhi, India; 12 International Zinc Association, New Delhi, India; Feroze Gandhi Degree College, INDIA

## Abstract

Understanding the spatial spreading patterns of plant-available sulphur (S) (AS) and plant-available micronutrients (available zinc (AZn), available iron (AFe), available copper (ACu), available manganese (AMn) and available boron (AB)) in soils, especially in coastal agricultural soils subjected to various natural and anthropogenic activities, is vital for sustainable crop production by adopting site-specific nutrient management (SSNM) strategies. We studied the spatial distribution patterns of AS, AZn, AFe, ACu, AMn, and AB in cultivated soils of coastal districts of India using geostatistical approaches. Altogether 39,097 soil samples from surface (0 to 15 cm depth) layers were gathered from farm lands of 68 coastal districts. The analysis of soil samples was carried out for soil pH, electrical conductivity (EC), soil organic carbon (SOC) and AS, AZn, AFe, ACu, AMn, and AB. Soil pH, EC and SOC varied from 3.70 to 9.90, 0.01 to 7.45 dS m^-1^ and 0.02 to 3.74%, respectively. The concentrations of AS, AZn, AFe, ACu, AMn, and AB varied widely in the study area with their corresponding mean values were 37.4±29.4, 1.50±1.53, 27.9±35.1, 2.14±1.74, 16.9±18.4 and 1.34±1.52 mg kg^-1^, respectively. The coefficient of variation values of analyzed soil parameters varied from 14.6 to 126%. The concentrations of AS, AZn, AFe, ACu, AMn, and AB were negatively and significantly correlated with soil pH and positively and significantly correlated with SOC. The geostatistical analysis indicated stable, Gaussian and exponential best-fit semivariogram models with moderate to strong spatial dependence for available nutrients. The generated spatial spreading maps revealed different distribution patterns for AS, AZn, AFe, ACu, AMn, and AB. There were variations in spatial spreading patterns of AS, AZn, AFe, ACu, AMn, and AB in east- and west-coastal area. About 62, 35, 12, 0.4, 23 and 45% of the study area had deficiency of AS, AZn, AFe, ACu, AMn, and AB, respectively. The spatial spreading maps will be highly useful for SSNM in the cultivated coastal soils of the country. This study could also be used as a base for assessing spatial spreading patterns of soil parameters in cultivated coastal areas of other parts of the world.

## Introduction

Earth has 620,000 km of coast line [1]. Around ¼ of the total population of the world resides in the coastal area (within a distance of 100 km from the coast line) and they predominantly depend upon agriculture and allied activities for livelihood [2]. India has approximately 7,500 km of coastline along Arabian sea and Bay of Bengal [3]. Both the coastal area and agriculture is very often adversely affected mainly by changes in salinity, tidal process, water stress, water logging and different anthropogenic activities. This leads to land degradation due to several reasons including soil nutrients deficiencies and reduced agricultural productivity [4, 5]. Therefore, efficient management of soil nutrients in coastal area is needed for higher agricultural productivity and better livelihood of the coastal population [6].

Depletion of phyto-available nutrients, henceforth referred as available nutrients (AN), in agricultural soils of different areas due to adoption of poor agricultural practices is an important reason for land degradation globally [7–11]. Along with nitrogen, phosphorus and potassium, deficiencies of AN like available sulphur (AS) and available micronutrients (available zinc (AZn), available iron (AFe), available copper (ACu), available manganese (AMn) and available boron (AB)) have been reported in various crops and soils world-wide [12–19]. The information pertaining to extent of AS, AZn, AFe, ACu, AMn, and AB deficiencies in various agricultural soils is limited. The availability of nutrients in soils is dependent on soil and crop types, and soil properties such as pH, electrical conductivity (EC), and soil organic carbon (SOC) content [14, 20]. Soil pH and SOC influence solubility and complexation of nutrients with organic acids in soil solution [21], whereas soil EC has been proved to relate with many soil properties influencing soil productivity [22, 23].

The knowledge about spatial spreading of AS, AZn, AFe, ACu, AMn, and AB and their associated soil properties is useful for devising site-specific S and micronutrients management strategies for better crop production [11, 24]. Various researchers studied spatial spreading of AN in soils for effective nutrient management [25–29]. Coastal soils of India are having various soil fertility constrains, adversely affecting crop production, due to geological setting, sediment load, cyclonic disturbances and varied agricultural management practices [30, 31]. The cultivated soils of coastal India are subjected to various natural and anthropogenic activities leading to variations in soil parameters including AS, AZn, AFe, ACu, AMn, and AB. This necessitates the need for better understanding of spatial spreading of AN for adoption of site- and/or area-specific nutrient management options for sustainable crop production. The spatial spreading of AS, AZn, AFe, ACu, AMn, and AB in coastal soils of India is expected to be high, and the information in this regard is limited [27, 32, 33].

Geostatistical technique is the useful and efficient one for assessing spatial spreading of soil parameters in farm, catchment, zonal and regional scales [34–37]. This technique predicts the values of the soil parameters at unsampled points considering spatial correlation of sampled points [38, 39]. This also models the spatial patterns of soil parameters through semivariogram analysis and prepare distribution maps by interpolation kriging. We carried out the present study with the hypothesis that spatial spreading of AS, AZn, AFe, ACu, AMn, and AB in cultivated soils of coastal India is high. The study aimed at to assess the status of soil pH, EC, SOC, AS, AZn, AFe, ACu, AMn, and AB and to prepare the spatial spreading maps of AS, AZn, AFe, ACu, AMn, and AB in cultivated soils of coastal districts of India, using geostatistical technique.

## Materials and methods

### Study area

**Fig 1:**
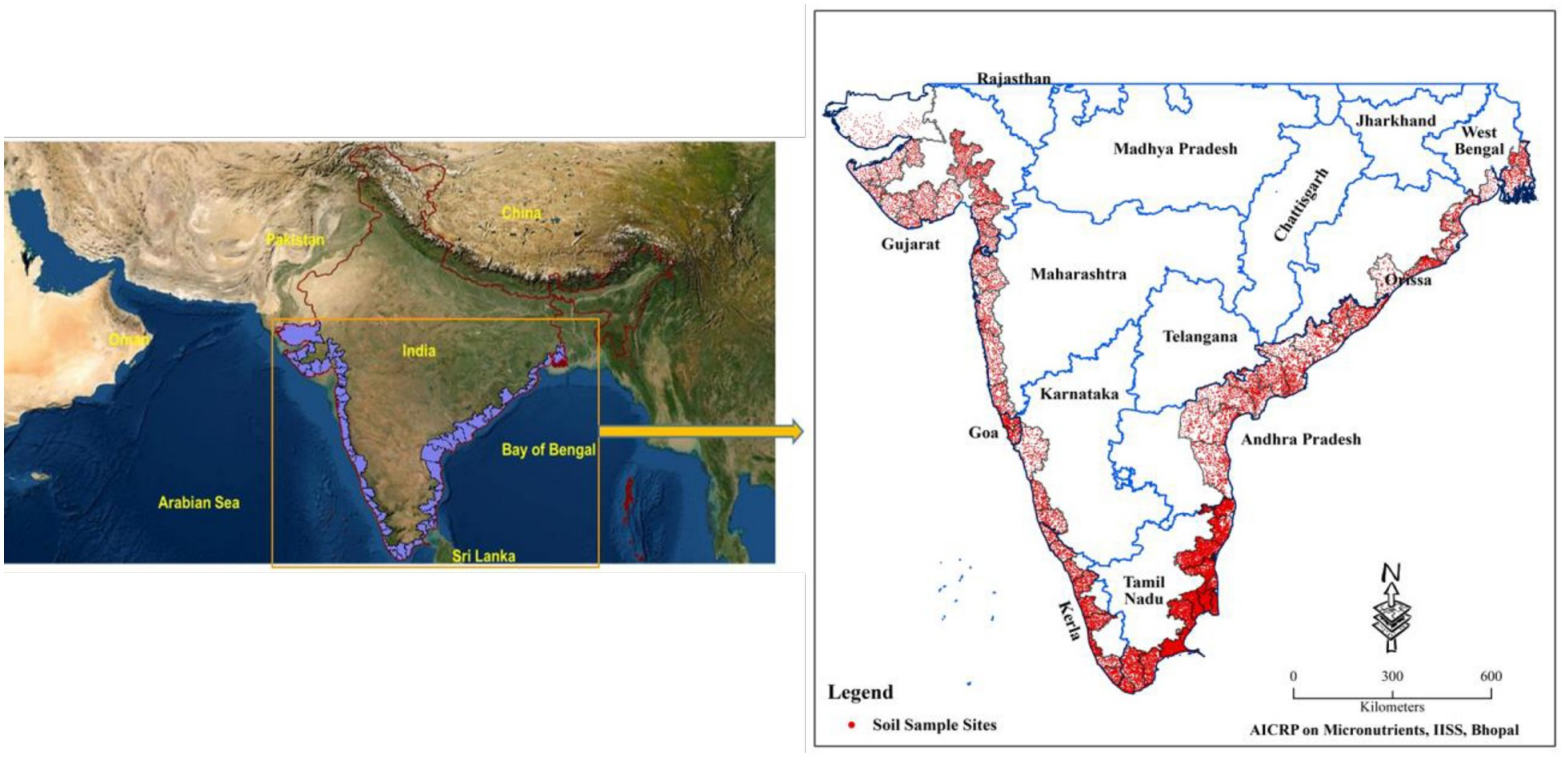
Soil sample locations in study area.

The soil samples were obtained from the cultivated lands of 68 coastal districts (Table 1), spreading across 9 different states of India (lying at 8.04° to 23.54° N latitude, 68.31° to 89.04° E longitude) (Fig 1), under a national project entitled “All India Coordinated Research Project on Micro and Secondary Nutrients and Pollutant Elements in Soils and Plants”. The study districts extend from West Bengal state in the eastern part (east coast) to Gujarat state in the western part (west coast) of the country. The districts experience hot and subhumid climate in east coast to hot semi-arid climate in west coast. The districts receive varied amount of average annual precipitation (AAP). Coastal districts of Gujarat, Kerala and Odisha state receives < 400, > 2500 and ≈1500 mm AAP, respectively [40]. Soils in the districts vary from east coast to west coast. Soils belong to Inceptisols, Entisols, Vertisols, Alfisols, Ultisols, and Aridisols orders with sandy-loam, clay-loam, clay, sandy-loam, loam, sandy-clay-loam, silty-clay-loam texture [41]. The important cultivated crops in the study districts are rice, pulses (food legumes), maize, cotton, sugarcane, oil seed, vegetables sand plantation crops like coconut and arecanut [42]. However, the detailed information related to area, production and yield of important crops of different districts of various states of India is available elsewhere [43].

**Table 1:** Coastal districts of various states in India.

State	Districts	State	District
Gujarat	Ahmedabad	Tamil Nadu	Chennai
	Anand		Cuddalore
	Bharuch		Kancheepuram
	Bhavnagar		Kanyakumari
	Jamnagar		Nagapattinam
	Junagadh		Ramanathapuram
	Kutch		Thiruvarur
	Navsari		Tirunelveli
	Porbandar		Thiruvallur
	Devbhoomi Dwarka		Thoothukudi
	Morvi		Villupuram
	Gir Somnath		Pudukkottai
	Amreli		Thanjavur
	Surat	Andhra Pradesh	East Godavari
	Valsad		Guntur
Maharashtra	Greater Mumbai		Krishna
	Raigad		Nellore
	Ratnagiri		Prakasam
	Sindhudurg		Srikakulam
	Suburban Mumbai		Visakhapatnam
	Thane		Vizianagaram
	Palghar		West Godavari
	Dadar Nagar Havel (Union territory)	Odisha	Baleshwar
Goa	North Goa		Bhadrak
	South Goa		Ganjam
Karnataka	Dakshin Kannada		Jagatsinghpur
	Udupi		Kendrapara
	Uttar Kannada		Puri
Kerala	Alappuzha	West Bengal	East Medinipur
	Ernakulam		North 24 Parganas
	Kannur		South 24 Parganas
	Kasaragod		
	Kollam		
	Calicut		
	Malappuram		
	Thiruvananthapuram		
	Thrissur		

### Soil sampling and analysis

Altogether 39097 surface (0–0.15 m depth) soil samples were obtained using a hand-held auger, from the cultivated lands of coastal districts during April to June months of 2016 to 2018 by adopting stratified random sampling procedure [44]. Prior to the collection of soil samples, due approval was obtained from the land owners. A hand-held global positioning system was used for recording the geographical coordinates of each sample point (provided as S1 Dataset). Each composite soil sample was obtained from 3–4 subsamples for small land holding (<1 ha), 6–7 subsamples for medium land holding (1–3 ha) and 9–10 subsamples for large land holding (>3 ha), to minimize sampling effect [45]. The soil samples were processed by air-drying in a dust-free environment and by removing stones and debrises. Subsequently, the samples were ground and sieved (using a 2 mm sieve) and kept in plastic jars for analysis.

Soil-water suspension (1:2.5 w/v) was used for estimation of soil pH and EC [46]. The SOC content and AS were estimated by wet-oxidation method [47] and 0.15% calcium chloride extraction method [48], respectively.

The concentrations of AZn, AFe, ACu and AMn were estimated after DTPA extraction [49] and measurement of metal concentrations in the extracts using atomic absorption spectrophotometer (AAS) (Make and model: Varian AA 240Z & GTA 120, USA, with the detection limits for metals varying from 0.01–0.05 mg L^-1^). The concentration of AB was measured using hot-water extraction method [50] and colour development by azomethine-H method. The intensity of developed colour was measured by spectrophotometer (Make and model: Shimadzu UV-1800, Japan).

### Quality control and assurance

The analysis of metallic micronutrients in soil samples was carried out using AAS. All the glass wares in the laboratory, used for micronutrient analysis, were sequentially washed with detergent solution, tap water, dilute nitric acid solution and double distilled water. The standards for metals (Merck, Germany) were utilized for standardization and calibration. The precision of the AAS was checked by carrying out calibrations at 50 samples interval.

### Data analysis

Using SAS 9.2 software package [51], the descriptive statistics of studied soil parameters were obtained. The relations among the studied soil parameters were evaluated by Pearson’s correlation coefficient analysis. The datasets pertaining to AS, AZn, AFe, ACu, AMn, and AB were checked for normal distribution [52], prior to geostatistical analysis, and data transformations were carried out. The semivariogram (Eq 1) analysis was carried out using ArcGIS 10.6 software (Esri, Redlands, California, United States) for characterizing spatial structure of AS, AZn, AFe, ACu, AMn, and AB [39]. The semivariogram draws a plot between the variance (of spatially separated points of each dataset) and the lag distance. The semivariogram at h distance interval is γ(h). The sample pair value at h distance interval is N(h). The symbols z(xi) and z(xi+h) denote the sample points separated by a distance h.


$$\gamma (\text{h})=\frac{1}{2\text{N}\left(\text{h}\right)}\sum_{\text{i}=1}^{\text{N}\left(\text{h}\right)}{\left[\text{z}\left(\text{xi}\right)-\text{z}\left(\text{xi}+\text{h}\right)\right]}^{2}$$ (1)

The best fitted semivariogram models were chosen by cross-validation and depending upon the value of root mean square error (RMSE) (Eq 2).

$$\text{RMSE}=\sqrt{\frac{1}{\text{n}}\sum_{\text{i}=1}^{\text{n}}{\left[\text{z}\left(\text{xi},\text{yi}\right)-\text{z}*\left(\text{xi},\text{yi}\right)\right]}^{2}}$$ (2)

Where, z(xi, yi) is observed value, z _*_ (xi, yi) is predicted value and n is number of observations.

The widely used technique of ordinary kriging (OK) was used in order to develop distribution maps. OK estimates the values at unsampled locations based on optimal, unbiased and linear estimation [53]. It is built on the assumptions of second-order stationary and spatial autocorrelation. The accuracy of interpolated maps was verified by goodness-of-prediction-criterium (G value expressed in %) [35]. The positive G value indicates more accuracy of interpolated map from the samples than the area average.

## Results

### Overall variability of soil parameters

Soil pH varied from 3.70 to 9.90 with mean value of 6.98 (Table 2). About 73% soil samples had pH ≥6.5. Soil EC and SOC content varied from 0.01 to 7.45 dS m^-1^ (mean 0.75 dS m^-1^) and 0.02 to 3.74% (mean 0.62%), respectively. About 25 and 35% samples had EC values <0.25 dS m^-1^ and ≥0.25 to <0.50 dS m^-1^, respectively. Soil organic carbon in 41% samples were in the range of ≥0.25 to <0.50%. The CV values were 14.6% for soil pH, 123% for EC and 78.8% for SOC. The concentration of AS varied from 0.60 to 156 mg kg^-1^ (mean 37.4 mg kg^-1^). About 21% samples had ≤15.0 mg kg^-1^ of available S. The mean concentrations of AZn (varied from 0.07 to 9.54 mg kg^-1^), AFe (varied from 0.85 to 273 mg kg^-1^), ACu (varied from 0.01 to 13.8 mg kg^-1^) and AMn (varied from 0.35 to 154 mg kg^-1^) were 1.50, 27.9, 2.14 and 16.9 mg kg^-1^, respectively. About 35% samples had AZn concentration of ≤0.6 mg kg^-1^. The concentration of AB varied from 0.01 to 12.8 mg kg^-1^, with the mean value of 1.34 mg kg^-1^. About 17% samples had AB concentration of ≤0.50 mg kg^-1^. The CV values of AS, AZn, AFe, ACu, AMn, and AB followed the order: AFe (126) > AB (113) > AMn (109) > AZn (102) > ACu (81.3) > AS (78.5).

**Table 2:** Descriptive statistics of soil parameters of cultivated areas of coastal India.

Soil Properties	Minimum	Maximum	Mean	SD	CV (%)	Skewness	Distribution
pH (1:2.5)	3.70	9.90	6.98	1.02	14.6	-0.53	-
EC (dS m^-1^)	0.01	7.45	0.75	0.37	123	5.86	-
SOC (%)	0.02	3.74	0.62	0.49	78.8	3.06	-
AS (mg kg^-1^)	0.60	156	37.4	29.4	78.5	1.24	Transformed
AZn (mg kg^-1^)	0.07	9.54	1.50	1.53	102	2.36	Transformed
AFe (mg kg^-1^)	0.85	273	27.9	35.1	126	3.04	Transformed
ACu (mg kg^-1^)	0.01	13.8	2.14	1.74	81.3	1.53	Transformed
AMn (mg kg^-1^)	0.35	154	16.9	18.4	109	2.96	Transformed
AB (mg kg^-1^)	0.01	12.8	1.34	1.52	113	3.53	Transformed

Note: SD = standard deviation, CV = coefficient of variation, EC = electrical conductivity, SOC = soil organic carbon, AS = available sulphur, AZn = available zinc, AFe = available iron, ACu = available copper, AMn = available manganese, AB = available B.

### Relationships among of soil parameters

Pearson’s correlation coefficient analysis highlighted the positive and significant relation of soil pH with AS (r = 0.060 p < 0.01) and negative and significant (r = -0.031 to -0.337 p < 0.01) relation with available AZn, AFe, ACu, AMn and AB (Table 3). Soil EC was positively and significantly related with available AS (r = 0.146 p < 0.01), AZn (r = 0.091 p < 0.01), AFe (r = 0.012 p < 0.05), ACu (r = 0.103 p < 0.01) and AMn (r = 0.079 p < 0.01). SOC content was negatively and significantly related with AS (r = -0.047 p < 0.01) and positively and significantly (r = 0.047 to 0.182 p < 0.01) related with AZn, AFe, ACu, AMn and AB. Available S was positively and significantly related with AZn (r = 0.036 p < 0.01), ACu (r = 0.078 p < 0.01), AMn (r = 0.056 p < 0.01) and AB (r = 0.317 p < 0.01). There were positive and significant relations among the studied available micronutrients in soils of coastal area. A pair of parameters having strong positive relations is expected to display similar distribution pattern. Whereas, a pair of parameters having strong negative relations is expected to display mirror image of spatial distribution pattern.

**Table 3:** Relationships among the soil parameters of cultivated areas of coastal India.

**Variables**	**pH**	**EC**	**SOC**	**AS**	**AZn**	**AFe**	**ACu**	**AMn**	**AB**
pH	1.000								
EC	0.079**	1.000							
SOC	-0.264**	-0.017**	1.000						
AS	0.060**	0.146**	-0.047**	1.000					
AZn	-0.229**	0.091**	0.156**	0.036**	1.000				
AFe	-0.337**	0.012*	0.182**	0.000	0.291**	1.000			
ACu	-0.134**	0.103**	0.125**	0.078**	0.295**	0.344**	1.000		
AMn	-0.183**	0.079**	0.124**	0.056**	0.321**	0.293**	0.344**	1.000	
AB	-0.031**	-0.005	0.047**	0.317**	0.072**	0.118**	0.185**	0.283**	1.000

Note: EC = electrical conductivity, SOC = soil organic carbon, AS = available sulphur, AZn = available zinc, AFe = available iron, ACu = available copper, AMn = available manganese, AB = available B.

* and ** denote significant at p < 0.05 and p < 0.01 respectively.

### Spatial variability of available S and micronutrients

**Fig 2:**
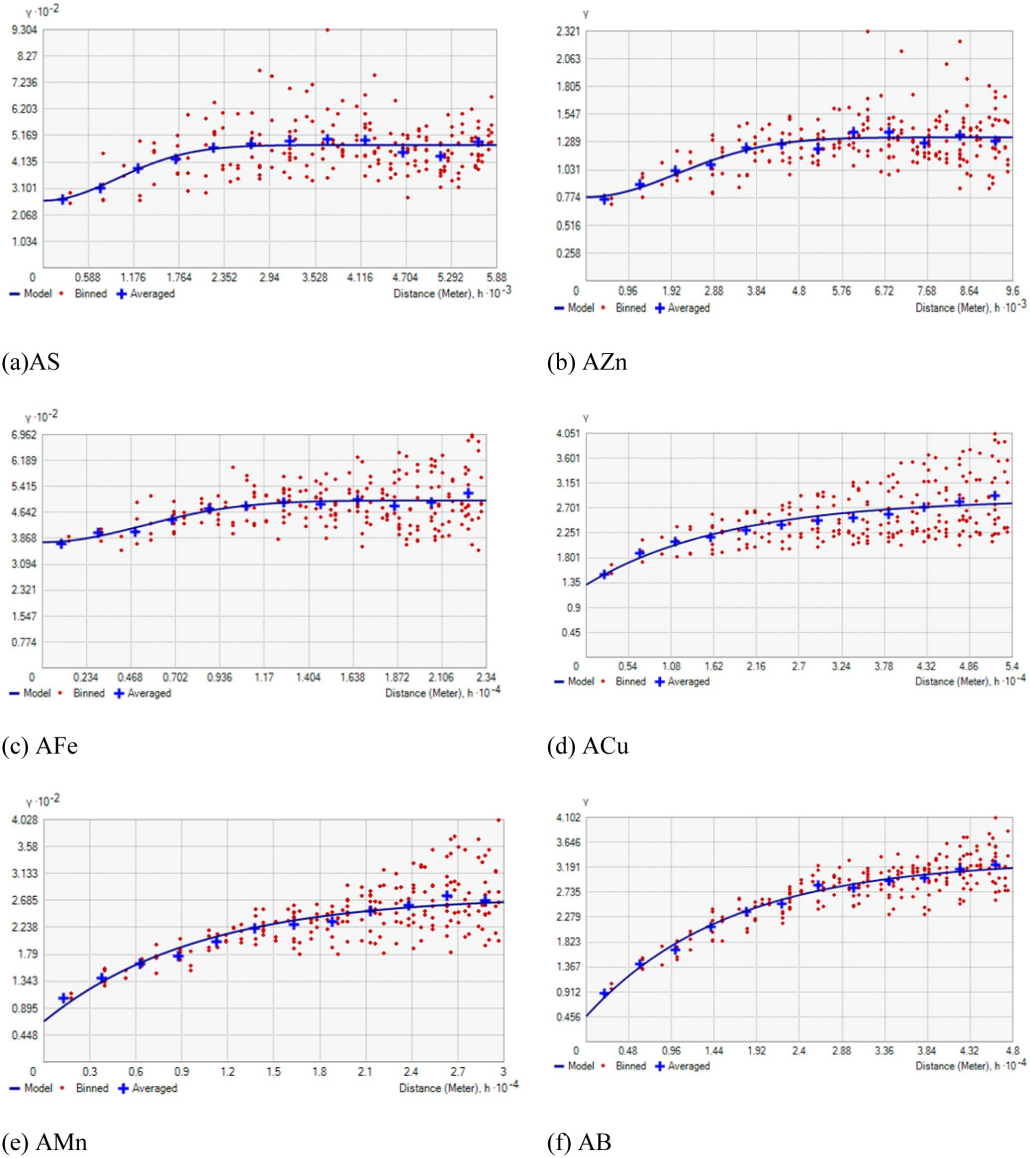
Semivariograms of available nutrients in study area. AS = available sulphur, AZn = available zinc, AFe = available iron, ACu = available copper, AMn = available manganese, AB = available B.

The semivariogram parameters of AS, AZn, AFe, ACu, AMn, and AB in coastal area were different (Table 4, Fig 2). Available sulphur had stable best-fit model. Available Zn and AFe had Gaussian best-fit model and ACu, AMn and AB had exponential best-fit model. The nugget values of AS, AZn, AFe, ACu, AMn, and AB were 0.47 to 375. AS, AFe and AMn had higher nugget values. The spatial dependence was moderate for AS, AZn and ACu and strong for AFe, AMn and AB. The range values for available nutrients varied from 2328 m (for AS) to 54000 m (for ACu).

**Table 4:** Semivariogram attributes of soil parameters of cultivated areas of coastal India.

Soil parameter	Model	Nugget	Partial sill	Sill	Nugget/Sill	Range (m)	Spatial dependence	RMSE	G (%)
AS (mg kg^-1^)	Stable	262.74	216.79	479.53	0.5479	2328	Moderate	18.380	51
AZn (mg kg^-1^)	Gaussian	0.7780	0.5551	1.3331	0.5836	5016	Moderate	1.0729	42
AFe (mg kg^-1^)	Gaussian	375.36	124.51	499.87	0.7509	12884	Strong	22.666	37
ACu (mg kg^-1^)	Exponential	1.3122	1.5506	2.8628	0.4583	54000	Moderate	1.1289	46
AMn (mg kg^-1^)	Exponential	67.486	208.85	276.336	0.2442	30000	Strong	11.280	51
AB (mg kg^-1^)	Exponential	0.4676	2.8514	3.319	0.1408	48000	Strong	0.6149	49

Note: **A**S = available sulphur, AZn = available zinc, AFe = available iron, ACu = available copper, AMn = available manganese, AB = available B, RMSE = root mean square error, G = Goodness-of-predict criterium.

**Fig 3:**
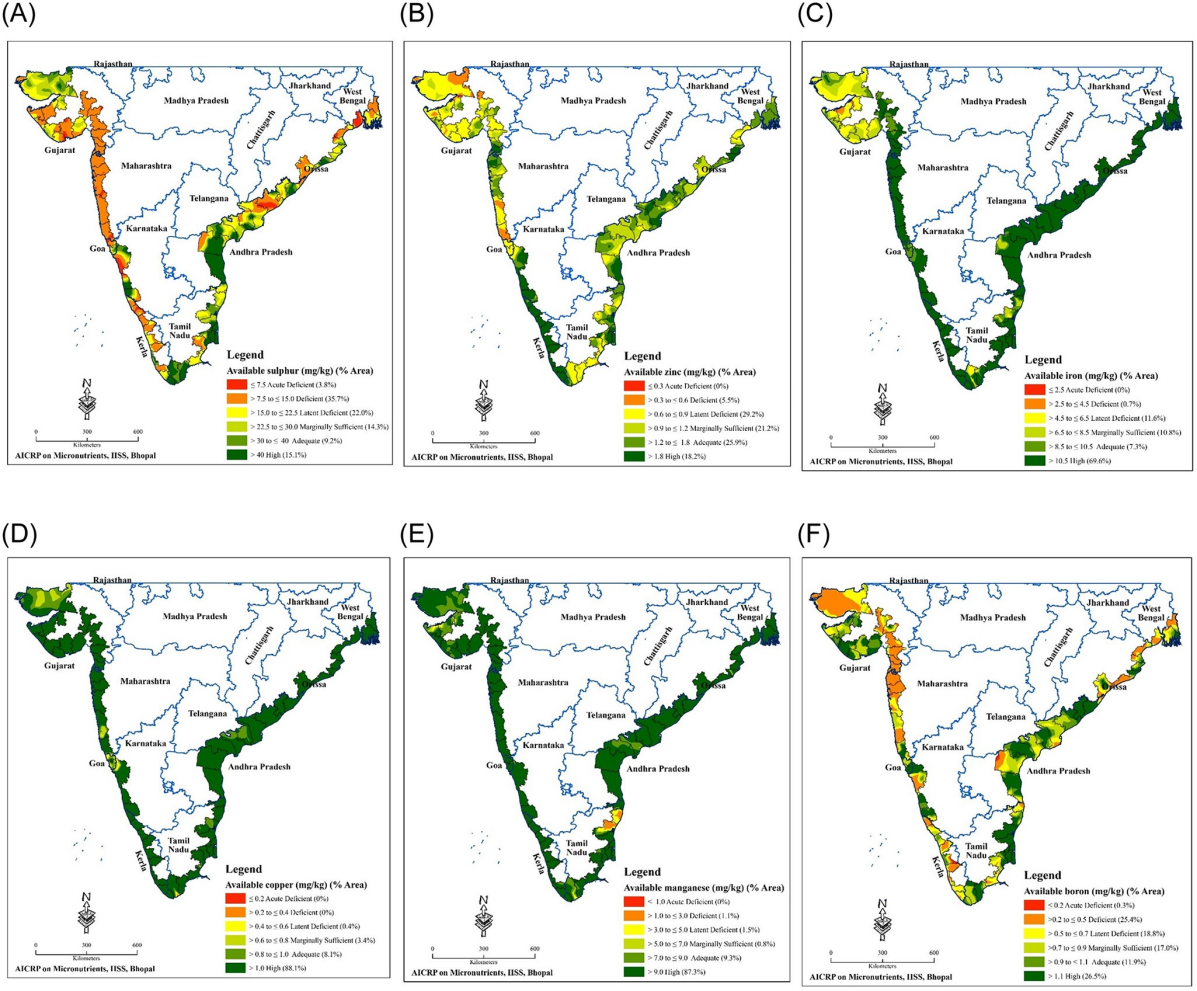
Distribution maps of available nutrients in study area.

Interpolation by OK produced distribution maps of soil available S and micronutrients with different distribution patterns (Fig 3). According to the classification proposed by Shukla and Behera [54] for Indian soils, the concentration of AS was ≤7.5 mg kg^-1^ (acute deficient), >7.5 to ≤15.0 mg kg^-1^ (deficient) and >15.0 to ≤22.5 mg kg^-1^ (latent deficient) in 3.80, 35.7 and 22.0% area, respectively. The higher portion of area in the west coast had AS concentration of ≤22.5 mg kg^-1^ compared to the portion of area in the east coast. The distribution map of AZn exhibited AZn concentration of ≤0.30 mg kg^-1^ (acute deficient), >0.30 to ≤0.60 mg kg^-1^ (deficient) and >0.60 to ≤0.90 mg kg^-1^ (latent deficient) in 0.0, 5.50 and 29.2% of area, respectively (as per the categories outlined by Shukla and Tiwari (2016) for Indian soils). Whereas, 21.2, 25.9 and 18.2% area had AZn value >0.90 to ≤1.20 mg kg^-1^ (marginally sufficient), >1.20 to ≤1.80 mg kg^-1^ (adequate) and >1.80 mg kg^-1^ (high), respectively. Major portion of the area in northern part of west coast and whole of east coast had AZn concentration of ≤0.90 mg kg^-1^. The concentrations of AFe in 0.0, 0.70 and 11.6% area were ≤2.50 mg kg^-1^ (acute deficient), >2.50 to ≤4.50 mg kg^-1^ (deficient) and >4.50 to ≤6.50 mg kg^-1^ (latent deficient), respectively. The major part of northern portion of west coast had AFe concentration of ≤6.50 mg kg^-1^. About 0.40% area had ACu concentration of >0.40 to ≤0.60 mg kg^-1^ (latent deficient). AMn concentrations were ≤1.00 mg kg^-1^ (acute deficient), >1.00 to ≤3.00 mg kg^-1^ (deficient) and >3.00 to ≤5.00 mg kg^-1^ (latent deficient) in 0.00, 1.10 and 1.50% area, respectively. About 0.30, 25.4 and 18.8% area had AB concentration in the range of ≤0.20 mg kg^-1^ (acute deficient), >0.20 to ≤0.50 mg kg^-1^ (deficient) and >0.50 to ≤0.70 mg kg^-1^ (latent deficient), respectively.

## Discussion

### Overall variability of soil parameters

The variations of soil pH (from highly acidic (3.70) to alkaline (9.90), EC (from 0.01 dS m^-1^ (non-saline) to 7.45 dS m^-1^ (saline)) and SOC (from 0.02 to 3.74%) were wide (Table 2). In line with our findings, Kharal et al. [55] reported the variations in soil pH and organic matter content in soils of Nepal under different land uses. The variations of soil pH, EC and SOC the study area is ascribed to the effects of the soil types, prevailing climatic conditions, and adoption of different crop-management practices. Soils of the study area are mainly deltaic alluvium [3]. However, the differences of soil properties in different parts of the study area are due to variations in nature of parent material and relief [41]. The wide variability in soil EC level in surface soils of the study area is because of differences in soil types, levels of precipitation, ground water level and intrusion of brackish water through creeks [56]. The variations in SOC status in the study area is attributed to differences in organic matter addition through crop residue and organic manure; and variations in prevailing climatic conditions [37]. The CVs followed the order EC (123%) > SOC (78.8%) > pH (14.6%). The lowest CV value for soil pH is attributed to logarithmic transformation of the hydrogen ion concentration. Similar CV values for pH, EC and SOC were also recorded in cultivated acid soils of India [57]. The concentrations of available AS, AZn, AFe, ACu, AMn and AB varied widely in the study area with their corresponding mean values 37.4±29.4, 1.50±1.53, 27.9±35.1, 2.14±1.74, 16.9±18.4 and 1.34±1.52 mg kg^-1^, respectively (Table 2). The CVs for AS, AZn, AFe, ACu, AMn and AB varied from 78.5 to 126%. This variation in concentrations of AS, AZn, AFe, ACu, AMn and AB and non-normal distribution of datasets are attributed to the nature of soils and various soil-crop managements including application of S and micronutrients containing fertilizers. Similarly, Shukla et al. [29] reported variations in concentrations of AS, AZn, AFe, ACu, AMn and AB with CV values varying from 57.2 to 85.7% in Indian part of Indo-Gangetic Plain. Behera et al. [11] also reported variations in concentrations of available AS, AZn, AFe, ACu, AMn and AB with CV values varying from 57.5 to 75.0% in Narmada River basin area of India. In soils of Nepal, Shrestha et al. [58] reported the variations in AZn and AB concentration due to change in crop rotations and thereby soil-crop management practices.

### Relationships among soil parameters

Soil pH was positively correlated with AS and negatively correlated with AZn, AFe, ACu, AMn and AB in coastal area of India. This indicates increase in AS and decrease in AZn, AFe, ACu, AMn and AB concentrations with increase in soil pH of the study area. This is because soil pH influences forms of soil nutrients and thereby affecting nutrients availability [21]. Khadka et al. [59] also recorded positive relationship between soil pH and AS in soils of western Nepal. Negative relations of soil pH with available cationic micronutrients (AZn, AFe, ACu and AMn) were reported by various researchers [60, 61]. Positive relation of soil EC with AS, AZn, AFe, ACu, AMn and AB in the study area supports the fact that EC is the indirect estimator of soil fertility level influencing crop yield. Positive relation of SOC with AS, AZn, AFe, ACu, AMn and AB reveals that AS, AZn, AFe, ACu, AMn and AB concentration increases with increase in SOC level in coastal area of India. This is attributed to influence of SOC on solubility and availability of studied nutrients. Positive relation of AS with AZn, ACu, AMn and AB and the positive relations among the studied micronutrients indicated their similar distribution pattern in the coastal area.

### Spatial spreading of available S and micronutrients

Understanding the spatial spreading patterns and generation of spatial spreading maps of soil nutrients help in site-specific soil nutrient management for sustainable production of crops [11, 28, 62]. The geostatistical analysis highlighted stable (for AS), Gaussian (for AZn and AFe) and exponential (for ACu, AMn and AB) best-fit models (Table 4, Fig 2). Similarly, Tesfahunegn et al. [35] and Shukla et al. [63] who reported various best fitted semivariogram models for different soil nutrients in a catchment area of Ethiopia and Himalayan Region of India., respectively. The values of nugget and sill of semivariogram highlight the amount of variance due to error from different sources and the variance from the observations by a distance, respectively. The higher nugget and sill values for AS, AFe and AMn (in comparison with ACu and AB) indicate the inability of the sampling distance to capture spatial dependence. This needs to be taken care while designing future sampling plans. Depending upon nugget/sill ratios, the spatial dependence is classified as strong (nugget/sill ratio ≤0.25), moderate (nugget/sill ratio >0.25 to ≤0.75) and weak (nugget/sill ratio >0.75) [38]. Available S, AZn and ACu had moderate spatial dependence. The strong spatial dependence for AFe, AMn and AB is ascribed to the soil properties. The moderate spatial dependence for AS, AZn and ACu is because of combined effect of soil properties and different soil-crop managements. The range values indicate the estimation of spatial extension in which autocorrelation exists between the samples. The higher range values reveal the impact of both natural and anthropogenic features on these soil parameters to a larger distance. Similarly, Shukla et al. [29] reported the range value of 13,115 to 60,000m for different soil parameters in Indian part of Indo-Gangetic Plain.

The spatial spreading maps for AS, AZn, AFe, ACu, AMn and AB of coastal agricultural soils of India, generated using OK interpolation, exhibit different distribution patterns (Fig 3). About 4, 36 and 22% area lying both in east and west coastal region were acute deficient, deficient and latent deficient in AS, respectively. Depending upon the level of S deficiency in different parts of the area, S-management options like addition of S-carriers for varied soil-crop situations need to be adopted. The spatial distribution map for AZn exhibited deficiency and latent deficiency in 5.50 and 29.2% area, respectively. Proper Zn management in these areas need to be prioritized for better crop yield and quality. Depending upon the levels of AZn, various Zn fertilizer doses, Zn-efficient crops and other Zn management strategies may be adopted in different areas [64–66]. About 12, 0.40 and 3% of the study area were deficient (acute deficient + deficient + latent deficient) in AFe, ACu and AMn, respectively. The distribution map revealed 0.30, 25 and 19% area with acute deficient, deficient and latent deficient in AB, respectively. The kriged distribution maps developed in this study are different from the online micronutrients maps available in farmers’ portal (www.farmer.gov.in/soilfertilitymaps.aspx) and soil health card (www.soilhealth.dac.gov.in) portal of India.

The results of our study revealed that there are differences in spatial spreading of AS, AZn, AFe, ACu, AMn and AB between the east and the west coast. This is attributed to the impact of different natural and anthropogenic factors. The east coast has extensive coastline with thicker alluvial sediments whereas, west coast has narrow coastline and having crystalline sediments [56]. This geological set-up influences hydrology of east and west coast. Moreover, the east coast has huge sediment load because of east-flowing rivers and it experiences frequent cyclonic disturbances. Further, the differences in prevailing temperature, rainfall, cropping pattern and crop management practices in east and west coast influence distribution of available S and micronutrients [31, 67]. Therefore, it is advisable to adopt site-specific S and micronutrients management options for sustainable crop production, by understating their spatial spreading patterns and considering local conditions and availability of resources. The developed distribution maps exhibiting heterogeneity in distribution pattern of AS, AZn, AFe, ACu, AMn and AB in cultivated soils of coastal districts of India, could be highly useful for site-specific management of these nutrients. Proper nutrient management will help in maintaining or improving soil health, and hence plant growth and agricultural productivity in the study area. The utility of these distribution maps could further be enhanced if the farmers and farm managers get acquainted with the characteristics of studied soil parameters, and plan site-specific management options including variable rate of input application for sustaining crop production with available resources. Further, the knowledge of spatial spreading pattern of AS, AZn, AFe, ACu, AMn and AB could be used by fertilizers industries, planners and policy makers for production, supply and delivery of right kind of S and micronutrients fertilizers for sustainable crop production in the coastal districts of the country.

## Conclusion

The study revealed the wider variations in soil pH, EC, SOC, AS, AZn, AFe, ACu, AMn and AB concentrations, with CV values of 14.6 to 126%, in cultivated soils of coastal districts of India. Available S, AZn, AFe, ACu, AMn and AB were differently correlated among themselves and with soil pH, EC and SOC. Available S and micronutrients had stable, Gaussian and exponential semivariogram models with moderate and strong spatial dependence. The spatial distribution patterns of available AS, AZn, AFe, ACu, AMn and AB highlighted the necessity for adoption of site-specific soil-crop management options for sustaining crop production in the study area. Similar investigations may be carried out in other coastal cultivated soils of the world for better comprehension of the spreading pattern of available soil nutrients, and adoption of effective site-specific soil-crop management options. This will help in obtaining economically viable and environmentally feasible crop yield. Additionally, the studies regarding spatial spreading of soil nutrients may be conducted by considering the associated soil and climatic parameters as co-variables for better understanding.

## Supporting information

S1 Dataset (RAR)
